# Synthesis and recycling of the mycobacterial cell envelope

**DOI:** 10.1016/j.mib.2021.01.012

**Published:** 2021-04

**Authors:** Katherine A Abrahams, Gurdyal S Besra

**Affiliations:** Institute of Microbiology and Infection, School of Biosciences, University of Birmingham, Edgbaston, Birmingham, B15 2TT, UK

## Abstract

*Mycobacterium tuberculosis* (*Mtb*), the causative agent of the disease tuberculosis, is a recognised global health concern. The efficacy of the current treatment regime is under threat due to the emergence of antibiotic resistance, directing an urgent requirement for the discovery of new anti-tubercular agents and drug targets. The mycobacterial cell wall is a well-validated drug target for *Mtb* and is composed of three adaptive macromolecular structures, peptidoglycan, arabinogalactan and mycolic acids, an array of complex lipids and carbohydrates. The majority of the enzymes involved in cell wall synthesis have been established, whilst studies directed towards the mechanisms of remodelling and recycling have been neglected. This review briefly describes mycobacterial cell wall synthesis, and focuses on aspects of remodelling and recycling, thus highlighting opportunities for future research.

## Highlights

• The unique mycobacterial cell wall is a layered structure of carbohydrates and lipids.
• The architecture and biosynthesis of the cell wall have largely been elucidated.
• Increasing evidence indicates that each cell wall layer is remodelled and recycled.
• There are opportunities to discover new essential enzymes in cell wall metabolism.
• Cell wall metabolism is a validated source of targets for tuberculosis drug discovery.

## Graphical abstract

**Figure:**
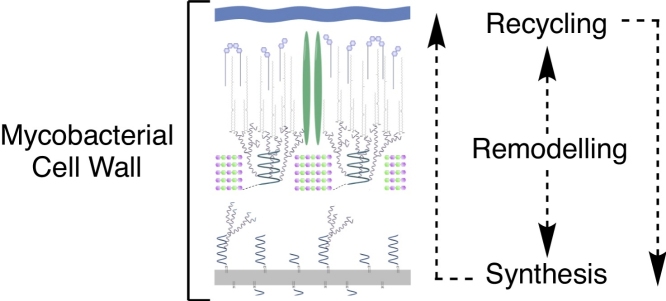

**Current Opinion in Microbiology** 2021, **60**:58–65

This review comes from a themed issue on **Special section on bacterial cell wall synthesis**

Edited by **Jean-François Collet** and **Angelika Gründling**

For complete overview of the section, please refer to the article collection, “Special section on Bacterial cell wall synthesis”

Available online 18th February 2021

**https://doi.org/10.1016/j.mib.2021.01.012**

1369-5274/© 2021 The Author(s). Published by Elsevier Ltd. This is an open access article under the CC BY license (http://creativecommons.org/licenses/by/4.0/).

## Introduction

*Mycobacterium tuberculosis* (*Mtb*), the pathogen responsible for tuberculosis (TB), is a leading cause of global mortality, contributing to approximately 1.4 million deaths in 2018 [1]. This worldwide burden has directed an impetus towards innovations in diagnostics, new therapies and healthcare provisions. However, the emergence of multi-drug resistant (MDR) and extensively drug resistant (XDR) strains of *Mtb* threatens these advances, driving the demand for further research into the biochemistry and pathogenicity of *Mtb*, with a view to discover novel anti-tubercular drugs and targets [2]. A defining characteristic of all mycobacteria is their cell envelope, which is a validated drug target of a number of first-line and second-line TB therapies [3]. As a result, it has been the subject of intensive research over the past two decades. Developments in genomic and molecular techniques have enabled the majority of the structural elements and biosynthetic pathways of the *Mtb* cell wall to be resolved. The most recent evidence has revealed that mycobacteria have the machinery to recycle their cell envelope, opening up the possibility of discovering a plethora of undefined enzymes [4^••^,^5^,^6^]. This review focuses on the current understanding of the structure, biosynthesis, remodelling and recycling of the mycobacterial cell envelope. The information gleaned from such studies could prove invaluable for drug discovery efforts in the on-going fight against *Mtb*.

## Mycobacterial cell wall architecture

**Figure 1:**
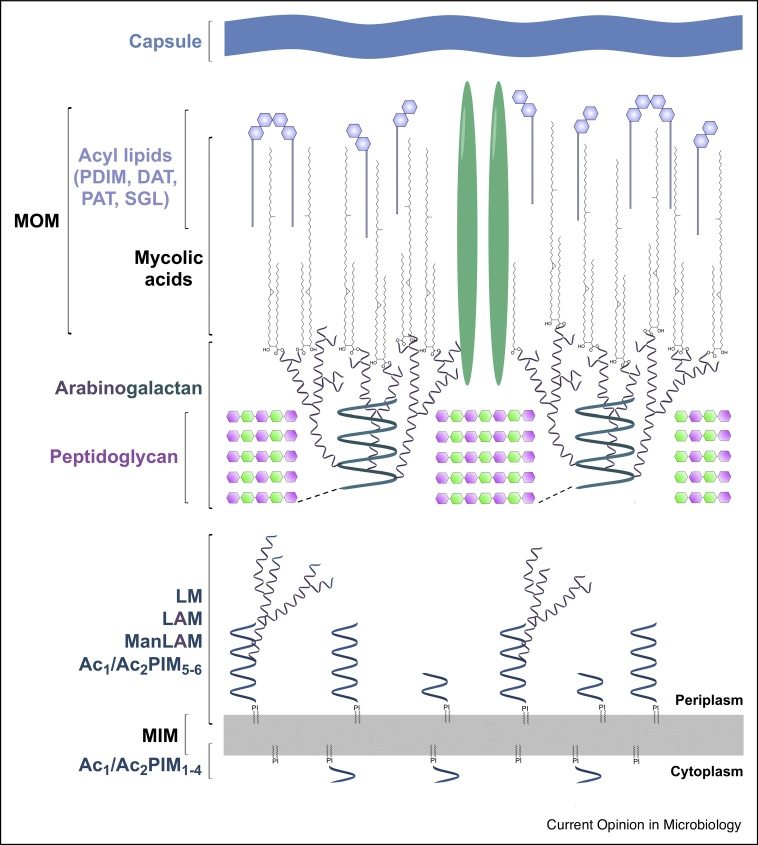
Mycobacterial cell wall architecture. A schematic representation of the mycobacterial cell wall, highlighting the key features. Abbreviations: mycobacterial inner membrane (MIM), mycobacterial outer membrane (MOM), phosphatidyl-*myo*-inositol mannosides, (PIMs, with acylation sites Ac_1_/Ac_2_), lipomannan (LM), lipoarabinomannan (LAM), mannosylated lipoarabinomannan (ManLAM), diacyl-trehalose (DAT), polyacyl-trehalose (PAT), phthiocerol dimycocerosate (PDIM), and sulfoglycolipid (SGL). An outer membrane protein has been included (green) to depict how solutes traverse the hydrophobic layer.

The mycobacterial cell wall is a unique macromolecular structure, sharing a few similarities with Gram-positive and Gram-negative bacteria. It is essential for cell viability, playing roles in structural integrity and pathogenicity [3]. The mycobacterial cell wall is intricate in architecture, composed of a core mycolyl-arabinogalactan-peptidoglycan complex (mAGP). Inner and outer-membranes are intercalated with non-covalently linked glycophospholipids, such as phosphatidyl-*myo*-inositol mannosides (PIMs), and the derivatives lipomannan (LM) and lipoarabinomannan (LAM), and other solvent extractable lipids, including diacyl-trehalose (DAT), polyacyl-trehalose (PAT), phthiocerol dimycocerosate (PDIM), and sulfoglycolipid (SGL) [3]. Proteins, such as porins, traverse the hydrophobic outer membrane, enabling the transport of hydrophilic solutes. A capsule of polysaccharides and proteins makes up the outermost layer. A schematic representation of this extensive cell envelope is shown in Figure 1.

## Peptidoglycan synthesis, remodelling and recycling

**Figure 2:**
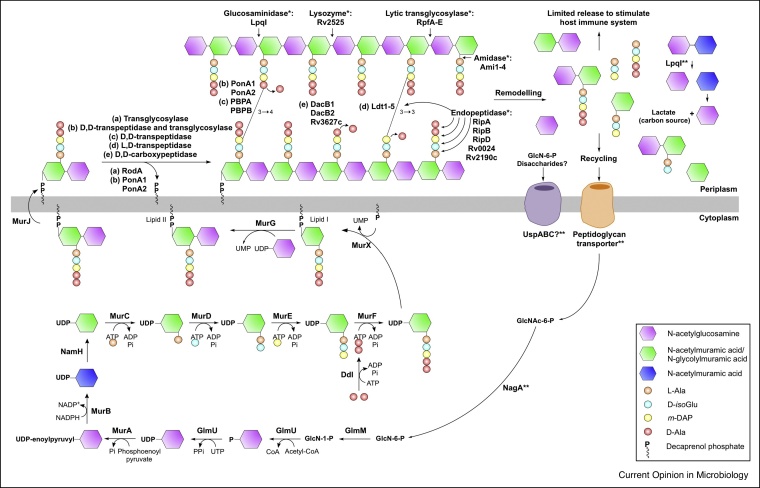
Peptidoglycan biosynthesis, remodelling and recycling. Peptidoglycan biosynthesis has been reviewed in detail [7]. Enzymes involved in peptidoglycan synthesis (no symbol), remodelling (*) and recycling (**) are shown in bold type. Question marks (?) indicate a predicted enzyme function.

Peptidoglycan is a polymer of alternating *N*-acetylglucosamine (GlcNAc) and *N*-acetylmuramic acid (MurNAc)/*N*-glycolylmuramic acid (MurNGlyc) residues, with peptide side chains that cross-link adjacent glycan chains. The biosynthesis of peptidoglycan, summarised in Figure 2, has long been established (reviewed in Maitra 2019) [7]. More recently, there has been a shift in focus towards elucidating: (i) the recruitment and modulation of enzymes at specific cellular locations during different growth phases and infection, (ii) the roles of enzymes in virulence, and (iii) the discovery of new inhibitors targeting peptidoglycan synthesis.

For instance, l,d-transpeptidation has gained much interest. Ldt2 [8, 9, 10, 11, 12, 13, 14], Ldt3 [15,16] and Ldt5 [17] have been biochemically characterised with known substrates and inhibitors. Furthermore, the spatial activity of l,d-transpeptidation has also been addressed; incubation of cells with Ldt-specific fluorescent substrate probes showed preferential labelling of the poles and septum before the sidewalls [18]. This intensive research highlights the importance of these enzymes, particularly from the perspective of inhibitor discovery [9,10,16,19]. Classical transpeptidation by Penicillin Binding Proteins (PBPs) has also received renewed interest, especially since the validated inhibition of the β-lactamase, BlaC [20, 21, 22, 23], which has re-established the potential of β-lactam antibiotics as a treatment for TB [24,25]. Many PBPs have redundant roles *in vitro*, however, they have critical roles for survival and virulence within the host. This is exemplified by PBPA and the transglycosylase RodA, which regulate cell length *in vitro* but are essential for survival and granuloma formation during infection [26].

Peptidoglycan is a dynamic structure and its integrity relies on co-ordination between enzymes involved in synthesis, remodelling and recycling, to enable a plethora of cellular processes, such as cell division, resuscitation and pathogenicity. The *Mtb* genome encodes for enzymes that are able to cleave the major covalent bonds in peptidoglycan, including glycosidases, amidases, endopeptidases and carboxypeptidases (Figure 2) [27]. The genetic multiplicity of the *Mtb* remodelling enzymes implies functional redundancy. For example, one of the five *Mtb* d,l-endopeptidases, RipA, is the major septal hydrolase in cell division, and has been shown to be non-essential for viability *in vitro*, where other endopeptidases, such as RipB, could compensate; depletion of *ripA* and *ripB* inhibits growth of *Mtb* [28^•^]. However, RipA has been shown to be essential for persistence in an infection model, indicating that the remodelling enzymes have very specific individual roles, not necessarily observed in *in vitro* studies, to allow for adaptations to environmental conditions. These specific functions are gradually being revealed for other remodelling enzymes. This includes the lytic transglycosylases, also known as resuscitation promotion factors (Rpfs). It has long been established that they promote resuscitation from dormancy, but recently they have also been shown to be involved in mycobacterial biofilm formation [29]. Following cleavage by the remodelling enzymes, there is limited release of the peptidoglycan fragments for immune system stimulation [4^••^].

Peptidoglycan recycling in mycobacteria has long remained debatable. Almost all mycobacteria lack homologues of the established recycling genes from other bacteria, with the exception of *nagA* and *nagZ*/*lpqI* [4^••^]. Biochemical analyses of the corresponding proteins, along with earlier efforts, such as the characterisation of UspC, the solute binding protein of the essential amino-sugar transporter UspABC, provide the first *bona fide* evidence to support mycobacterial peptidoglycan recycling [6]. The cytoplasmic mycobacterial NagA catalyses the deacetylation of *N*-acetylglucosamine-6-phosphate (GlcNAc-6-P) to glucosamine-6-phosphate (GlcN-6-P), which can be shunted back either into cell wall biosynthesis (GlmM and GlmU) or into glycolysis [5]. This suggests that uptake of the GlcNAc moiety of peptidoglycan does exist, but whether this is a fragment of peptidoglycan or its single sugar form is yet to be elucidated. Recent evidence has shown that the *N*-acetylglucosaminidase, LpqI, is capable of cleaving the glycosidic bond between GlcNAc–MurNAc fragments [4^••^]. It has been proposed that further metabolism of the MurNAc moiety liberates lactate, which can be used as a sole carbon source. Together, this information gives credence to peptidoglycan recycling in mycobacteria (Figure 2), opening up the potential to discover novel metabolic pathways.

## Arabinogalactan synthesis and remodelling

**Figure 3:**
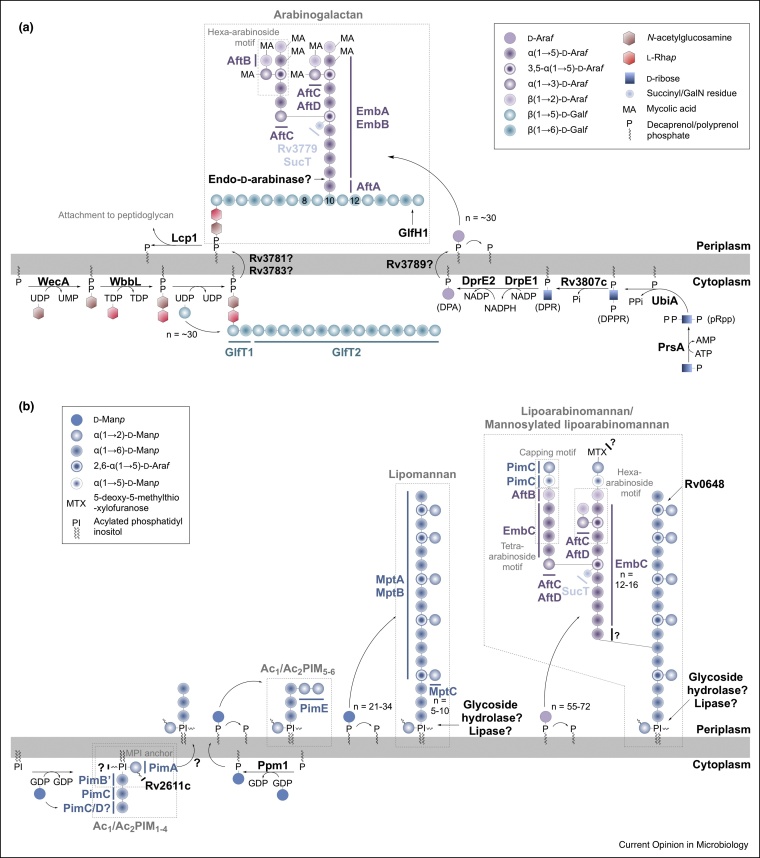
Arabinogalactan, PIMs, LM, LAM and ManLAM biosynthesis and remodelling. **(a)** The synthesis of arabinogalactan and **(b)** synthesis of the glycolipids, PIMs, LM, LAM and ManLAM. Arabinogalactan and glycolipid biosynthesis are the subjects of a recent comprehensive review [3]. Abbreviations: phospho-α-d-ribosyl-1-pyrophosphate (pRpp), decaprenol-1-monophosphate 5-phosphoribose (DPPR), decaprenol-1-phosphoribose (DPR), decaprenylphosphoryl-d-arabinose (DPA), d-arabinose in furanose ring form (d-Ara*f*), d-galactose in furanose ring form (d-Gal*f*), l-rhamnose in pyranose ring form (l-Rha*p*), d-mannose in pyranose ring form (d-Man*p*), phosphatidyl-*myo*-inositol mannosides, (PIMs, with acylation sites Ac_1_/Ac_2_).

Arabinogalactan, a branched heteropolysaccharide, is covalently attached to the peptidoglycan layer, together forming an integral part of the cell wall. The chemical architecture of arabinogalactan and its biosynthesis, as shown Figure 3, is described in a detailed review [3]. Until recently, with limited reports of an endo-d-arabinase [30,31], there was little evidence to support arabinogalactan remodelling and recycling. However, a recent study by Shen *et al.* has identified an exo-β-d-galactofuranose hydrolase, Rv3096, termed GlfH1, which hydrolyses the recurrent terminal β-(1,5) and β-(1,6)-galactofuranose linkages of the galactan chain of arabinogalactan [32^••^]. This evidence provides a basis for future research into arabinogalactan remodelling and recycling from a biochemical and structural perspective.

## Phosphatidyl-*myo*-inositol mannosides, lipomannan and lipoarabinomannan synthesis and remodelling

Embedded in the inner and outer membranes of the cell envelope are the glycolipids phosphatidyl-*myo*-inositol mannosides (PIMs), lipomannan (LM) and lipoarabinomannan (LAM). These major cell wall constituents exhibit immunomodulatory activities and also contribute to TB pathogenesis [33]. Their structures and biosynthesis have been reviewed extensively [3,34] and are detailed in Figure 3.

To date, there is little information in the literature regarding the remodelling and recycling of PIMs, LM and LAM. Studies have shown that differences in the ratios of these glycolipids can determine virulence and the outcome of an infection, which could be controlled at the synthetic level or by theoretical remodelling and recycling activities that are yet to be established [35]. The mannan and arabinomannan moieties of LM and LAM are major constituents of the mycobacterial capsule; arabinomannan has been detected in *Mtb in vivo* and *in vitro*, to varying degrees [36]. This suggests that there is an undefined enzyme responsible for releasing the polysaccharides from their respective lipid anchors.

The observed heterogeneity of mannan and arabinomannan chain lengths in capsular material, as well as in LM and LAM, suggests the presence of novel glycoside hydrolases. To support this notion, almost two decades ago, an enzyme, Rv0648, was shown to exhibit α-mannosidase activity [37]. Although no further information regarding this enzyme has been reported, an endo-α-(1-6)-d-mannanase has recently been characterized from *Bacillus circulans* [38^•^], and is used to degrade environmental sources of mannose polymers (from plants and fungi). This could further facilitate the discovery of similar enzymes in mycobacteria. The mannosidase activity, along with the endogenous arabinose activity previously discussed, suggests that at least some remodelling and recycling does exist, although it may not be extensive.

## Mycolic acid biosynthesis, remodelling and recycling

**Figure 4:**
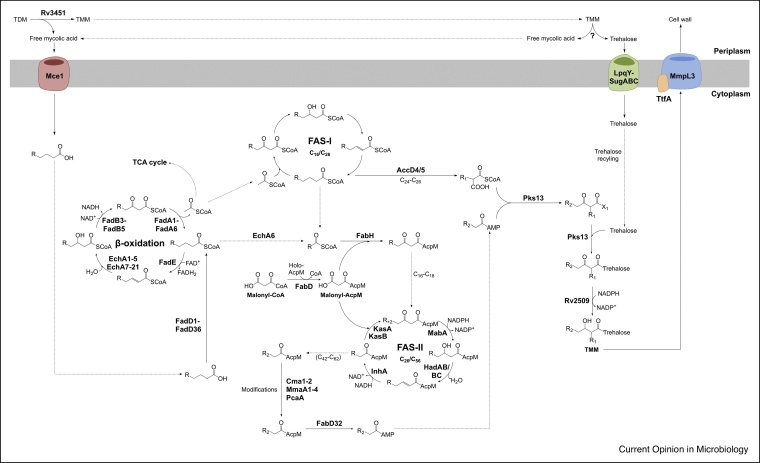
Mycolic acid biosynthesis and recycling. Intermediate chain-length fatty acids and mycolic acids can be recycled through the β-oxidation pathway and trehalose taken up and used directly in the synthesis of TMM/TDM (trehalose monomycolate/dimycolate). The synthesis of mycolic acids has been described in a detailed review [39].

Mycolic acids make up the outermost layer of the mycobacterial cell wall. These unique, long chain α-alkyl-β-hydroxy fatty acids are composed of a C_24_–C_26_ saturated α-chain and a meromycolate chain up to C_56_; the two chains are synthesised by two discrete pathways, FAS-I and FAS-II, as detailed in Figure 4 and reviewed in Batt *et al.* [39]. MmpL3, along with recently identified accessory proteins, including TtfA [40^•^], is responsible for the transport of mycolates across the membrane in the form of trehalose monomycolate (TMM). Mycolates are then attached to arabinogalactan by the mycolyltransferases of the Antigen 85 complex, or to another TMM, forming trehalose dimycolate (TDM).

The thickness of the *Mtb* cell wall changes during different phases of growth and infection, where remodelling and recycling of the impermeable lipid layer is important in the response to host defences, chemotherapeutic treatments and nutrient availability [41]. Consequently, structural and compositional differences are observed in mycobacteria grown *in vitro* and *in vivo*. This is exemplified by recent research on *Mycobacterium abcessus*, which shows that the cell surface lipids undergo significant remodelling under infection-relevant growth conditions [42]. Lipids are valuable carbon sources, and *Mtb* has the ability to uptake and metabolise host-derived fatty acids and cholesterol [43]. Similar to the cholesterol multi-protein importer Mce4, a related ABC-binding cassette transporter, Mce1, has been implicated in fatty acid import and recycling of mycolic acids [44]. Disruption of the *mce1* operon leads to increased *de novo* fatty acid biosynthesis [45] and free mycolic acids [46,47]. The *mce1* operon is repressed during the first eight weeks of infection in a mouse model [48], where nutrient starvation could then trigger Mce1 expression, enabling free mycolic acids to be used as a carbon source.

Following import, fatty acids can be shuttled to the β-oxidation pathway (Figure 4) to generate acetyl-CoA, which can then feed-back into mycolic acid biosynthesis, or into central metabolism in the tricarboxylic acid cycle. Recent evidence suggests that long-chain acyl-CoAs could bypass the β-oxidation pathway and be transferred directly to FAS-II biosynthesis using catalytically inactive (but classified as) enoyl-CoA hydratases, such as EchA6 [49]. Recycling in this way would enable significant energy conservation. For the recycling of free mycolic acids, they first need to be hydrolysed from either arabinogalactan or trehalose (TMM or TDM). A hydrolase of TDM, Rv3451, has been identified [50], which is induced during nutrient starvation, making its role in mycolic acid recycling plausible. This opens up the possibility of discovering a hydrolase of TMM, where the liberated trehalose can also be recycled by the LpqY-SugA-SugB-SugC ABC transporter, which is specific for the uptake of trehalose [51]. The ability of *Mtb* and mycobacteria to recycle these rich carbon sources contributes to their success in persistent infections and requires further investigation.

## Conclusion

Mycobacterial cell wall architecture and biosynthesis are largely elucidated and mechanistic insights into recycling are gradually becoming available. Importantly, available evidence shows for at least some genes, there are differences in essentialities and roles of the enzymes *in vivo* and *in vitro*. This information has, and will continue to prove instrumental in the development of treatment strategies against *Mtb*. Currently, two of the drugs in the front-line TB treatment regimen, isoniazid and ethambutol, target cell wall biosynthesis and are effective against active *Mtb* infections. Although, there are no reported drugs targeting cell wall recycling, this process is important for the viability of *Mtb* in persistent populations. Therefore, concomitantly targeting cell wall biosynthesis and recycling could be invaluable in TB treatment, inhibiting both active and latent forms of *Mtb*. To fulfil this aspiration, a collaborative response of pioneering methods in drug discovery combined with the comprehensive understanding of the pathogenicity and biochemistry of the microorganism, will prime the future response in this on-going battle against *Mtb*.

## Conflict of interest statement

Nothing declared.

## References and recommended reading

Papers of particular interest, published within the period of review, have been highlighted as:

• of special interest
•• of outstanding interest

## CRediT authorship contribution statement

**Katherine A Abrahams:** Conceptualization, Writing - original draft, Writing - review & editing, Visualization. **Gurdyal S Besra:** Conceptualization, Resources, Writing - original draft, Writing - review & editing, Visualization, Supervision, Project administration, Funding acquisition.
